# Prevalence of Long COVID Syndrome and its Association With Blood Group: A Cross-Sectional Study

**DOI:** 10.7759/cureus.53966

**Published:** 2024-02-10

**Authors:** Muhammad Abdurrahman Butt, Muhammad Areeb Abdullah, Mustafa Waseem, Hamna Ahmed, Amna Aamir, Rafia Asif, Hameed M Durrani

**Affiliations:** 1 College of Medicine, Shifa College of Medicine, Shifa Tameer-e-Millat University, Islamabad, PAK; 2 Department of Community Medicine, Shifa College of Medicine, Shifa Tameer-e-Millat University, Islamabad, PAK

**Keywords:** covid-19, coronavirus disease 2019, abo blood groups, fatigue, cross sectional studies, prevalence, blood group, long covid

## Abstract

Background: The coronavirus disease 2019 (COVID-19) virus pandemic rapidly spread across the globe since 2020. It was characterized by a number of acute signs and symptoms. There were, however, some new-onset signs and symptoms labelled as “Long COVID”. This study was conducted to study its prevalence and associations with blood group.

Methods: A retrospective analysis was conducted in Islamabad for patients diagnosed with COVID-19 in 2020-2021. Information was collected through an online and physical questionnaire regarding personal demographics, symptoms during and after COVID-19, and blood group. The data was analyzed using IBM SPSS Statistics for Windows, Version 25.0 (Released 2017; IBM Corp., Armonk, New York, United States).

Results:The study identified 196 participants out of which 48.5% were male and 51% were female, with a median age of 30. Most participants (62.2%) belonged to the Punjabi ethnicity. The mean BMI was 25.56 kg/m^2^. The majority of the study participants reported having blood group B (n=76) followed by A (n=52). Acute symptoms were experienced by 95.4% of participants, with fatigue being the most persistent symptom at four weeks (45.9%). After four weeks, 63.3% of participants reported new symptoms like hair loss. Females were found more likely to experience long COVID symptoms. The perceived severity of acute infection was significantly associated with long COVID symptoms (p=<0.01). There was no association found between long COVID and blood group (p=0.158).

Conclusion: There was no association found between long COVID and ABO blood groups. Females were more likely than males to experience long COVID. Long COVID was found to be more likely to develop in those with perceived severe acute infection, highlighting the need for further research regarding aggressive care from the onset of COVID-19 infection.

## Introduction

Novel coronavirus, severe acute respiratory syndrome coronavirus-2 (SARS-CoV-2) is a member of a family of coronaviruses that has caused the coronavirus disease 2019 (COVID-19). The first confirmed human infections were reported in December 2019 from Wuhan City, China, as cases of unexplained pneumonia. The infection spread quickly until it was no longer contained to Wuhan, with cases appearing in the United States and Europe. In January 2020, COVID-19 was declared a global health emergency by WHO and, shortly after, a pandemic in March 2020 [1]. The virus has since then wreaked havoc over the globe, with over 689,000,000 people infected and 6,800,000 deaths worldwide, as of June 4, 2023 [2].

The incubation period of the virus, that is the time between exposure to the virus and the appearance of symptoms, is on average four to five days but can be as long as 14 days. A wide range of symptoms has been reported in the past few years, with the most common being fever, chills, cough, shortness of breath, fatigue, myalgia and body aches, loss of smell and taste, nausea, vomiting, diarrhoea, sore throat, congestion, and runny nose [3-4]. Recovery is usually within a few weeks of the illness. These acute symptoms have been the sole focus of attention for a while, but over time there have been suggestions of a more persistent, long-lasting illness caused by the virus, which has since been termed “Long COVID” [5]. CDC uses the term post-acute COVID syndrome (PACS) to describe “health issues that persist more than four weeks after first being infected with the virus.”

The term has quickly gained traction amongst the general public, as reports of lingering and long-lasting symptoms months after recovery from COVID-19 continue to rise. The manifestations of this illness include symptoms such as persistent cough, myalgia, gastrointestinal disturbances, dyspnea, sleep difficulties, palpitations, anxiety, and depression [6-7]. Among these, one of the most prominent complaints in survivors of COVID-19 has been chronic fatigue [8]. Multiple studies have also looked into the multi-organ dysfunction that may be caused by COVID-19 [9-11].

A study conducted in Karachi has explored long-term COVID-19 infection and found persisting symptoms in patients even after recovery from COVID-19 [12]. A one-year follow-up study in Pakistan showed that symptoms like fatigue persisted when the patients came back for follow-up [13].

A study on the relationship between ABO blood groups and Long COVID infection severity shows that patients with AB blood group have persistent symptoms [14]. A large cohort study in Denmark, published in 2023, tried to explore the correlation between Long COVID and blood groups but no association was found [15]. To the best of our knowledge, no research has been carried out on this topic at both local and national levels in Pakistan. Therefore we decided to further study this association so that each individual can get personalised treatment related to the symptoms of their specific blood group post discharge from the hospital. This study aimed to find the prevalence of Long COVID symptoms in the population of Islamabad and also to determine any association with ABO blood groups.

This article was previously presented as a poster at the 12th Shifa Annual Scholars Day 2023 on August 29, 2023.

## Materials and methods

This was a cross-sectional study conducted in the capital city of Pakistan, Islamabad. After approval from the Shifa International Hospitals Ltd. Institutional Review Board & Ethics Committee (approval number: 345-21), data collection was initiated. Patients who were diagnosed with COVID-19 in 2020-2021 by polymerase chain reaction (PCR) were invited to be a part of this study and were part of the final analysis. 

The participants were selected by non-probability convenience sampling technique. Participants who gave their informed consent (n=196) were asked to fill out a physical or online (Google Forms; Google LLC, Mountain View, California, United States) questionnaire through which their demographic and biological details, symptomatic history including acute, persistent, and Long COVID, and the presence of co-morbidities were obtained. Symptoms occurring after a period of four weeks of being diagnosed with COVID-19 by PCR were considered symptoms of Long COVID. Participants who didn’t mention their blood group were excluded.

The data was analysed using IBM SPSS Statistics for Windows, Version 25.0 (Released 2017; IBM Corp., Armonk, New York, United States). The quantitative data was presented as means with standard deviations or medians with maximum and minimum values. The qualitative data was presented in frequencies and percentages. Pearson chi-square (Χ²) tests (p-value <0.05) were performed to find any association of Long COVID with age, BMI, gender, blood group, ethnicity, comorbidities and smoking status. It was also performed to find any association between the occurrence of Long COVID and the severity of acute infection.

## Results

**Figure 1:**
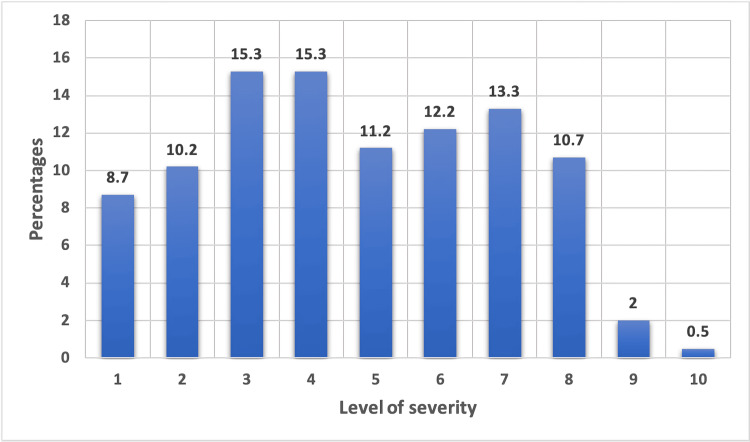
Severity of symptoms

There were a total of 196 participants in this study who fulfilled the inclusion and exclusion criteria and were part of the final analysis. There were 95 males (48.5%) and 100 females (51.0%) participants with one participant who did not mention his/her gender. The median age was 30 years ranging from 15 to 74 years. In this study, most participants (n=122) reported themselves as belonging to Punjabi ethnicity. The mean BMI of the participants was 25.56 kg/m^2^ ± 4.46 SD. Fifty-seven (29.1%) participants reported suffering from other illnesses of which 28 were hypertensive and only 53 were taking medicines for these other illnesses. The majority of the study participants reported having blood group B (n=76) followed by A (n=52). Only 25 participants (12.8%) were smokers (Table 1). On a scale of 1-10 with 1 being the least severe and 10 being the most severe, the majority of the participants reported the severity of their symptoms as 3 or 4 (15.3% each) (Figure 1). 

**Table 1:** Participants’ demographic and medical history

	Number of participants (n)	Percentages (%)
Blood group		
A	52	26.5
B	76	38.8
AB	19	9.7
O	49	25.0
Ethnicity		
Punjabi	122	62.2
Pakhtoon	18	9.2
Sindhi	2	1.0
Baloch	2	1.0
Kashmiri	14	7.1
Urdu-speaking	27	13.8
Gilgit Baltistani	5	2.6
Afghani	1	0.5
Missing	5	2.6
Gender		
Male	95	48.5
Female	100	51.0
Missing	1	0.5
Comorbidities		
Diabetes mellitus	16	8.2
Hypertension	28	14.3
Cardiovascular disease	3	1.5
Tuberculosis	0	0.0
Hepatitis B	0	0.0
Hepatitis C	2	1.0
Asthma	10	5.1
Cancer	2	1.0
Others	20	10.2
Medical treatment of comorbidities		
Diabetes mellitus	16	8.2
Hypertension	26	13.3
Cardiovascular disease	3	1.5
Tuberculosis	0	0.0
Hepatitis B	0	0.0
Hepatitis C	1	0.5
Asthma	9	4.6
Cancer	2	1.0
Others	15	7.7
Smoking		
Yes	25	12.8
No	169	86.2
Missing	2	1.0

**Figure 2:**
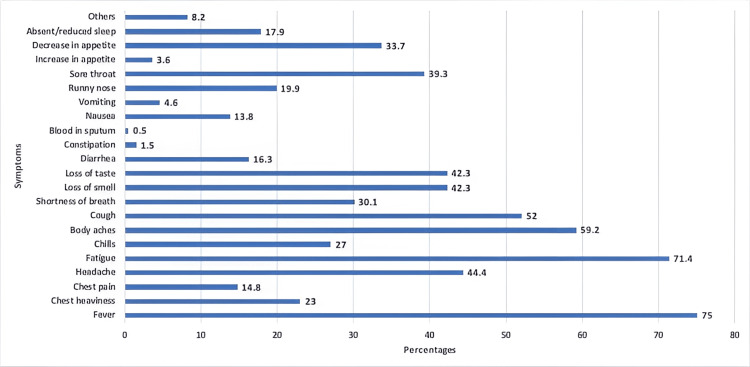
Prevalence of acute COVID-19 symptoms COVID-19: coronavirus disease 2019

**Figure 3:**
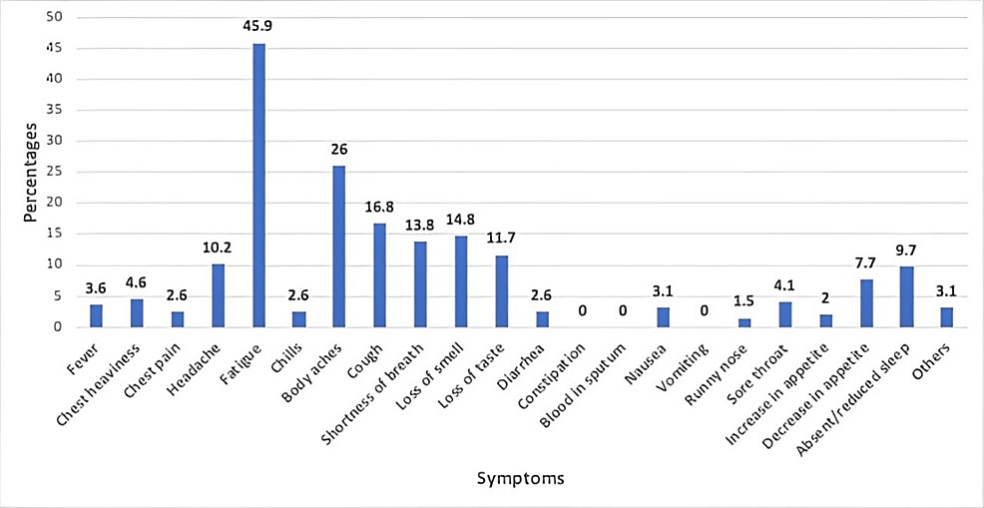
Prevalence of persistent COVID-19 symptoms COVID-19: coronavirus disease 2019

A total of 187 (95.4%) participants suffered from acute symptoms (within four weeks of diagnosis) which included fever (75.0%), fatigue (71.4%), body aches (59.2%), cough (52.0%), etc. (Figure 2). The majority of these participants (n=139) continued to experience various symptoms beyond four weeks with a prominent persistence of fatigue (45.9%) (Figure 3).

**Figure 4:**
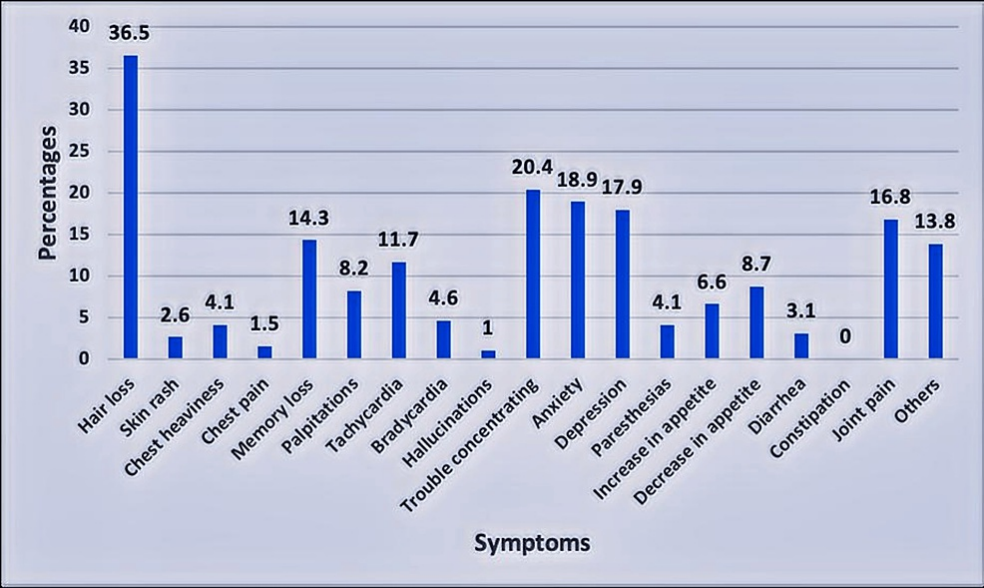
Prevalence of Long COVID symptoms

A total of 124 (63.3%) participants started to suffer from new symptoms after four weeks of diagnosis which included hair loss (36.5%), trouble concentrating on tasks (20.4%), anxiety (18.9%), depression (17.9%), and joint pains (16.8%) (Figure 4). Among these, 40 participants were taking medical treatment for these new symptoms (Table 2).

**Table 2:** Long COVID symptoms and use of medicines

	Number of Participants (n)	Percentages (%)
Long COVID symptoms (after 4 weeks)		
Hair loss	52	36.5
Skin rash	5	2.6
Chest heaviness	8	4.1
Chest pain	3	1.5
Memory loss	28	14.3
Palpitations	16	8.2
Tachycardia	23	11.7
Bradycardia	9	4.6
Hallucinations	2	1.0
Trouble concentrating	47	20.4
Anxiety	37	18.9
Depression	35	17.9
Paraesthesias	8	4.1
Increase in appetite	13	6.6
Decrease in appetite	17	8.7
Diarrhea	6	3.1
Constipation	0	0.0
Joint pain	33	16.8
Others	27	13.8
Medication use		
Hair loss	14	7.1
Skin rash	2	1.0
Chest heaviness	5	2.6
Chest pain	2	1.0
Memory loss	1	0.5
Palpitations	3	1.5
Tachycardia	3	1.5
Bradycardia	1	0.5
Hallucinations	0	0.0
Trouble concentrating	0	0.0
Anxiety	6	3.1
Depression	5	2.6
Paraesthesias	2	1.0
Increase in appetite	0	0.0
Decrease in appetite	3	1.5
Diarrhea	2	1.0
Constipation	0	0.0
Joint pain	14	7.1
Others	0	0.0

Gender was found to be associated with long COVID symptoms (Χ² (df=2, n=196)=11.303, p=0.010), with females being more likely to suffer from these symptoms (74 out of 100) as compared to males (50 out of 95). There was no association found with age (p=0.786), BMI (p=0.990), blood group (p=0.158), ethnicity (p=0.622), comorbidities (p=0.527), and smoking status (p=0.653). However, the perceived severity of acute COVID-19 infection was found to be significantly associated with occurrence of Long COVID symptoms (p=<0.001).

## Discussion

This study's results showed that Long COVID syndrome was prevalent in the population of Islamabad while no association with ABO blood group was determined.

COVID-19 has proven to be a peculiar condition. Fever was reported as the most common symptom in the acute phase of the illness, followed by fatigue, body aches and cough. After the resolution of the acute phase, many patients reported symptoms that either persisted long after other symptoms diminished or developed after the acute phase [12]. Our study's results correlated with previous studies that fatigue was the most common symptom that persisted among patients with COVID-19 [13]. This was followed by body aches and cough (Figure 3).

The most common symptom that developed after the acute phase, reported by over one-third of our patients, was hair loss, followed by anxiety, depression and joint pain (Figure 4, Table 2). This contrasts with a previous study that reported fatigue as the most prevalent symptom of Long COVID [16]. One of the proposed pathophysiologies of Long COVID is long-term tissue damage and pathologic inflammation, which could be driven by the persistence of the virus inside the body, immune dysregulation, or autoimmunity [17].

One significant finding of our study was the association between gender and Long COVID. Women were more likely to develop Long COVID compared to men, which is consistent with other studies that reported similar findings [17,18]. Other risk factors mentioned in the literature include having more than five early symptoms, early dyspnea, prior psychiatric disorders, and specific biomarkers such as D-dimers, C-reactive protein, and lymphocyte count [17]. A possible explanation for this in our clinical setting could be that symptoms like hair loss may be more noticeable in women and more likely to be reported, whereas, in men, hair loss may be more likely to be mistaken for androgenic alopecia.

In our study, the majority of patients reported the severity of their illness as mild. This could partly be explained by patient factors in our sample. Established risk factors for severe outcomes in COVID-19 infection include diabetes mellitus, hypertension, old age, chronic lung diseases, and heart, kidney, and liver diseases [19]. Our sample's median age was 30 years, and a low percentage of patients overall had comorbid conditions such as diabetes mellitus, hypertension, asthma, and cardiovascular diseases. Another established risk factor for severe outcomes in COVID-19 is smoking [20]. Only 12% of our patient sample were smokers, which could also explain why the majority of the patients experienced a mild illness.

Our study found no association between blood group and Long COVID syndrome. A study by Moslemi et al. also reported no association between blood group and Long COVID syndrome [15], while another study reported blood group AB being associated with patients having persistent symptoms [14].

Another significant finding of our study was the association between the severity of acute illness and Long COVID syndrome. People who had severe infections were more likely to develop either persistent symptoms or Long COVID syndrome. This concurs with previous literature that the severe acute phase of COVID-19 puts patients at a higher risk of developing Long COVID. A proposed mechanism would be that a severe disease process would lead to more cytokine release and an amplified inflammatory process, which would cause long-term organ damage that eventually culminates in the symptoms of Long COVID syndrome [21].

Further research is needed to determine the pathophysiology of Long COVID to better understand this condition. A suggestion to prevent or perhaps mitigate it could be to provide aggressive care from the start to patients admitted for COVID-19, particularly those with severe infections. Only further research can provide guidance in the future regarding this poorly understood condition.

One of the major limitations of our study was that participants had difficulty recalling many of the symptoms they experienced if they had contracted the virus a long time ago. Secondly, participants had described conditions like depression subjectively and were not subject to proper psychiatric evaluation. Furthermore, this study was limited to the population of Islamabad and to make it more generalised, further studies are required globally in a geographically widely-distributed area.

## Conclusions

Our study has shown that Long COVID is a common complication of acute COVID-19 infection. However, there was no association of Long COVID syndrome with the ABO blood group system found in our limited study of the Pakistani population. Furthermore, our study found that a significant proportion of patients who had developed Long COVID were females as compared to males. Our study also reported that patients who had experienced severe disease during acute disease were more likely to develop Long COVID.
